# AMF species do matter: *Rhizophagus irregularis* and *Funneliformis mosseae* affect healthy and PVY-infected *Solanum tuberosum* L. in a different way

**DOI:** 10.3389/fmicb.2023.1127278

**Published:** 2023-04-17

**Authors:** Edyta Deja-Sikora, Klaudia Werner, Katarzyna Hrynkiewicz

**Affiliations:** Department of Microbiology, Faculty of Biological and Veterinary Sciences, Nicolaus Copernicus University, Toruń, Poland

**Keywords:** *Solanum tuberosum* L., potato virus Y (PVY), *Rhizophagus irregularis*, *Funneliformis mosseae*, mycorrhiza level, oxidative stress, photosynthesis

## Abstract

Arbuscular mycorrhizal fungi (AMF) were documented to positively influence plant growth and yield, which is extremely important for the production of many crops including potato. However, the nature of the interaction between arbuscular mycorrhiza and plant virus that share the same host is not well characterized. In this study, we examined the effect of different AMF, *Rhizophagus irregularis* and *Funneliformis mosseae*, on healthy and potato virus Y (PVY)-infected *Solanum tuberosum* L. The analyses conducted included the measurement of potato growth parameters, oxidative stress indicators, and photosynthetic capacity. Additionally, we evaluated both the development of AMF in plant roots and the virus level in mycorrhizal plants. We found that two AMF species colonized plant roots to varying degrees (ca. 38% for *R. irregularis* vs. 20% for *F. mosseae*). *Rhizophagus irregularis* had a more positive effect on potato growth parameters, causing a significant increase in the total fresh and dry weight of tubers, along with virus-challenged plants. Furthermore, this species lowered hydrogen peroxide levels in PVY-infected leaves and positively modulated the levels of nonenzymatic antioxidants, i.e., ascorbate and glutathione in leaves and roots. Finally, both fungal species contributed to reduced lipid peroxidation and alleviation of virus-induced oxidative damage in plant organs. We also confirmed an indirect interaction between AMF and PVY inhabiting the same host. The two AMF species seemed to have different abilities to colonize the roots of virus-infected hosts, as *R. irregularis* showed a stronger drop in mycorrhizal development in the presence of PVY. At the same time, arbuscular mycorrhiza exerted an effect on virus multiplication, causing increased PVY accumulation in plant leaves and a decreased concentration of virus in roots. In conclusion, the effect of AMF-plant interactions may differ depending on the genotypes of both symbiotic partners. Additionally, indirect AMF-PVY interactions occur in host plants, diminishing the establishment of arbuscular mycorrhiza while changing the distribution of viral particles in plants.

## Introduction

Under specific environmental conditions, plant growth and physiological traits strongly depend on their interactions with symbiotic and/or pathogenic microorganisms and biotic factors such as viruses. Endophytes inhabiting plants can provide their host with some advantages to survive many environmental stresses (e.g., drought and salinity) and resist phytopathogens. Arbuscular mycorrhizal fungi (AMF), commonly found in soil, create symbiotic associations called arbuscular mycorrhiza (AM, which is a type of endomycorrhiza) with the roots of ~80% of land plants (Brundrett and Tedersoo, 2018). AMF, as biotrophs, depend on carbohydrates and lipids provided by plants for the completion of their life cycle. Host plants benefit from AMF mainly under low nutrient supply in soil (i.e., insufficient P and N availability), as AMF hyphae transfer minerals from the soil to plant roots (Bowles et al., 2018; Averill et al., 2019; Chowdhury et al., 2022). Furthermore, mycorrhizal networks extending outside the rhizosphere provide many more advantages to plants, including more efficient water acquisition, translocation of signaling compounds, and enhancement of plant defense mechanisms (Bitterlich and Franken, 2016). Hence, AMF are considered to function as plant bioprotectors and are increasingly used for plant pathogen control (Dey and Ghosh, 2022; Weng et al., 2022).

Endomycorrhizal fungi are essential for supporting soil ecosystem services. AMF used as biofertilizers were documented to positively influence plant growth and yield, which is extremely important for the production of many crops (Tang et al., 2022; Wu et al., 2022), including wheat (García de León et al., 2020), soybean (Marro et al., 2020), pepper (Guzman et al., 2021), and potatoes (Hijri, 2016). Potatoes, one of the most important crop plants worldwide, were reported to establish AM with different AMF species that are an inherent part of the agricultural soil microbiome, i.e., *Rhizophagus irregularis* (basionym *Glomus irregulare*, syn. *R. irregulare*), *Rhizophagus intraradices* (basionym *Glomus intraradices*), and *Funneliformis mosseae* (basionym *Endogone mosseae*, syn. *Glomus mosseae*) (Douds et al., 2007; Lone et al., 2015; Chifetete and Dames, 2020; Deja-Sikora et al., 2020). The application of AMF-based inocula in pot, greenhouse, and field experiments confirmed that arbuscular mycorrhiza can increase potato tuber biomass (Davies et al., 2005; Hijri, 2016; Lombardo et al., 2021) and enhance potato resistance to many different phytopathogens, e.g., *Rhizoctonia solani* (Yao et al., 2002) and *Phytophthora infestans* (Gallou et al., 2011). However, the nature of the interaction between AM and potato viruses, the prominent pathogens of potatoes, is not well characterized (Deja-Sikora et al., 2019). Sipahioglu et al. (2009) suggested that inoculation with *R. intraradices* induced more severe symptoms of disease in plants infected with potato virus Y (PVY). In contrast, in studies by Deja-Sikora et al. (2020), it was found that *R. irregularis* caused no exacerbation of PVY infection but reduced oxidative stress in plants impacted by the virus. Similarly, the results of other studies using different combinations of solanaceous host plants, AMF species, and virus groups/genotypes were also inconclusive (Shaul et al., 1999; Miozzi et al., 2011). Therefore, the role of AMF in the course of plant viral disease development remains unclear. Based on data published to date, it seems that AM consistently contributed to increased photosynthetic activity in virus-challenged plants (Sipahioglu et al., 2009; Miozzi et al., 2011; Deja-Sikora et al., 2020). In some investigations, improved photosynthetic parameters corresponded to a more severe impact of the virus and to elevated accumulation of viral particles in mycorrhizal plants (Miozzi et al., 2011). In other studies, despite higher photosynthesis levels, viral infection remained asymptomatic in AMF-colonized plants (Deja-Sikora et al., 2020). It was proposed that there may exist a different level of functional compatibility between symbiotic partners (measured as an amount of P transport by fungal hyphae to plant), which vary with the AMF species and host genotype (Ravnskov and Jakobsen, 1995; Duffy and Cassells, 2000; Singh et al., 2012; Yang et al., 2012). This means that specific host varieties may interact with AMF species in a different way (Fiorilli et al., 2022). Furthermore, host–AMF compatibility may be modulated by soil properties, showing that multiple factors influence the final effect of plant–fungus interactions (Duffy and Cassells, 2000). As this interesting phenomenon is not fully clarified, we decided to check how the same host interacts with two different AMF species, especially when the host is additionally impacted by the phytovirus.

The aim of this investigation was to compare the effect of two AMF species, i.e., *R. irregularis* and *F. mosseae*, on growth parameters, oxidative stress level, and photosynthetic capacity in potato cv. Pirol infected with PVY. The fungal species used in our study belong to different taxonomic groups (i.e., genera). We expected that the outcome of plant–AMF interactions would change because of different levels of mycorrhiza. Based on the obtained results, we wanted to evaluate which of the two AMF species provides more benefits to the host impacted by viral pathogens. Additionally, we intended to examine the interaction between AMF and PVY inhabiting the same plant, as all biotic factors sharing the same host may influence not only the host but also each other. We hypothesized that PVY would contribute to worsening host quality. This was expected to disturb arbuscular mycorrhizal development in plant roots resulting in (i) decreased AMF colonization intensity and (ii) lowered influence of AMF on plant condition. At the same time, we assumed that arbuscular mycorrhiza would affect virus multiplication and distribution in plant organs.

## Materials and methods

### Experimental design

Potato plants used in the experiment originated from *in vitro* cultures. After acclimatization, each plant was transferred to a pot with sterile sand, and the experiment started. Pots were put together in pairs consisting of one PVY-free (PVY–) and one PVY-infected (PVY+) potato plant. Plants from one pair were placed into the same isolated tray compartment. The roots of both plants were separated from each other by a stainless steel grid (pore size 30 μm) replacing one of the pot walls and covering the pot bottom. Grid pores were sufficiently large to allow AMF hyphae to pass through (Supplementary Figure 1). Control plants were grown without fungi, while the other plants were inoculated with spores of AMF. Thus, we received six experimental variants, i.e., two controls marked as P^PVY−^ and P^PVY+^, and four inoculated with two species of AMF (*F. mosseae* and *R. irregularis*) marked as P^PVY−^+ Fm, P^PVY+^+ Fm, P^PVY−^+ Ri, and P^PVY+^+ Ri. Each pair of variants was analyzed in four biological replicates consisting of six technical replicates of plants.

### Biological material

Potato virus Y-infected and virus-free plantlets of potatoes (*Solanum tuberosum* cv. Pirol) were grown *in vitro* on solid MS medium under the following conditions: light intensity of 200 μmol m^2^ s^−1^, long photoperiod of 16 L/8 D, and temperature of 17.5–18°C. Two species of AMF, i.e., *R. irregularis* and *F. mosseae*, were purchased from INOQ GmbH (Schnega, Germany). AMF spores were prepared for inoculation according to the instructions provided by the manufacturer. The PVY strain used in the experiments belonged to the N:O/N-Wilga group and caused systemic infection in the host (Deja-Sikora et al., 2020).

### Pot experiment

**Figure 1:**
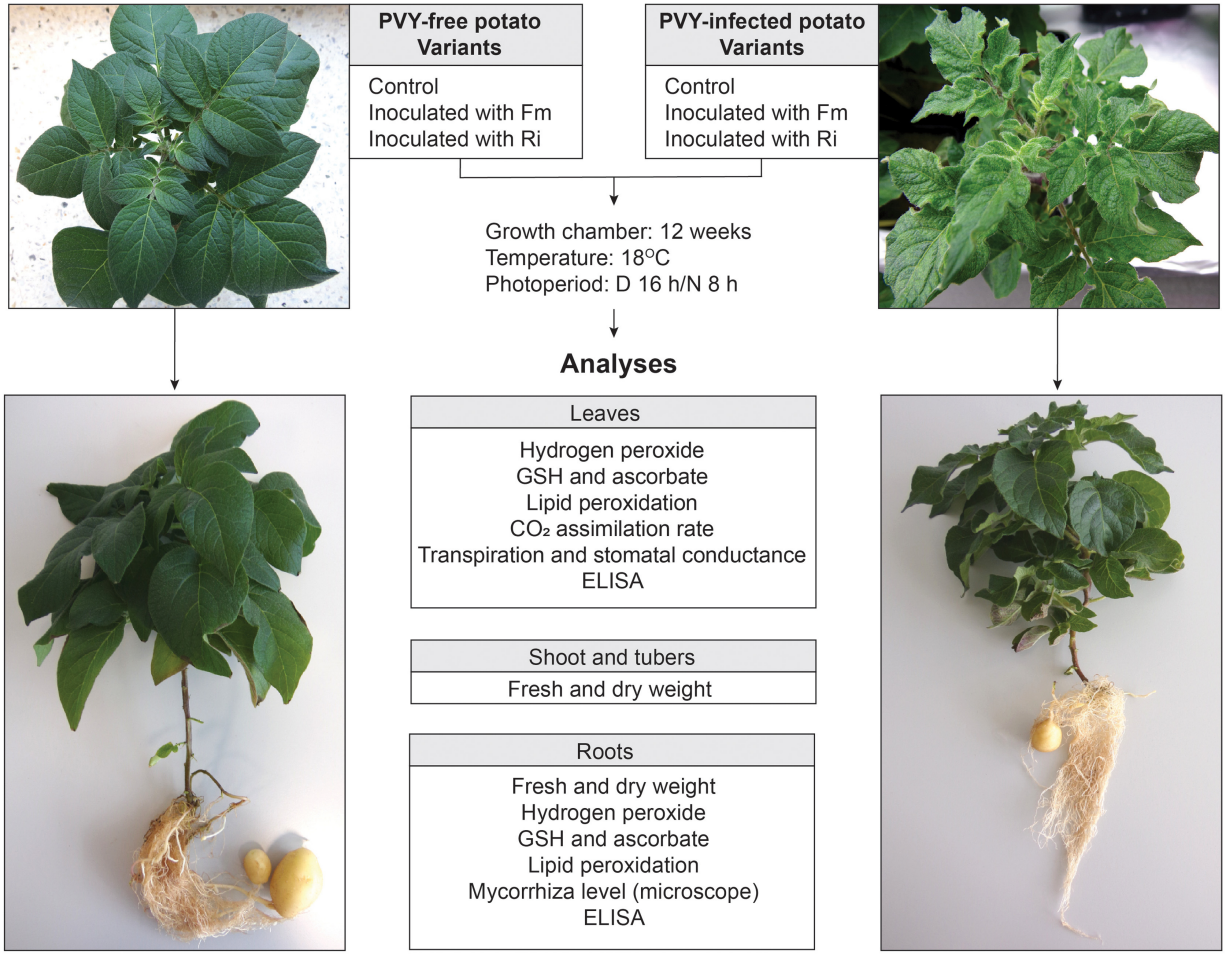
Experimental design. The model consisted of healthy and PVY-infected potato cv. Pirol inoculated with two AMF species—*Rhizophagus irregularis* and *Funneliformis mosseae*. Control plants were grown without mycorrhizal fungi. After 12 weeks of incubation plants were uprooted and analyzed according to the included scheme.

Two-week-old potato plantlets with developed roots (at least one, 1-cm long) were removed from the MS medium and rinsed with sterile water until the roots were free of agar. Plantlets were transferred to 0.5-liter pots filled with sand that were sterilized two times in an autoclave (121°C, 45 min). The experiment consisted of six variants, as shown in Figure 1. The inoculation of plants with AMF was performed by applying 50 mg of powder inoculum (number of propagules–250 million/kg) directly onto the root during plantlet transfer from *in vitro* culture to the sand. Plantlets were watered with 50 ml of Hoagland medium with a 10-fold decreased concentration of phosphate (P 0.1 mM) and covered with a foil tent to enable acclimatization. The adaptation period lasted 3 weeks. The foil tent was perforated in the last week to provide air exchange. After acclimatization, plants were watered three times per week with Hoagland medium with increasing P concentrations from 0.15 mM in the fourth week to 0.5 mM in the ninth week. Then, the concentration of 0.5 mM P was maintained until the end of the experiment. Plants were grown for 12 weeks.

### Plant growth parameters

Twelve-week-old potato plants were carefully removed from the substrate, and roots were rinsed with tap water. The growth parameters measured during sampling included the fresh weight of shoots, roots, and tubers, shoot length, and the number of nodes and tubers. After 48 h of incubation at 75°C, the dry weights of shoots, roots, and tubers were determined.

### Photosynthetic gas exchange

The rate of photosynthetic gas exchange in leaves was examined in the last week of the experiment with a Ciras-3 Portable Photosynthesis System (PP Systems, Amesbury, MA, USA). The measurements were conducted using a PLC3 universal leaf cuvette (leaf area 4.5 cm^2^) and a PLC3 LED light unit. During measurements, the following parameters were applied: ambient temperature (~20°C), sunlight-simulating RGBW settings (red: 38%, green: 37%, blue: 25%, and white: 0%), and CO_2_ reference–390 μmol mol^−1^; H_2_O reference–70%; PAR internal—the series of three consecutive values of 1,500, 1,800, and 2,200 μmol m^2^ s^−1^. For each plant, the photosynthetic activity of the fourth leaf was analyzed for 10–15 min. When the stabilization of the CO_2_ assimilation rate (A—μmol CO_2_ m^2^ s^−1^) was obtained at every next PAR value, 5–10 measurements were recorded.

For the A parameter, two variables, reflecting the change in the amount of assimilated CO_2_, were calculated according to the formulas: dA1 = A_1,800_-A_1,500_ and dA2 = A_2,200_-A_1,500_ to compare plant reactions to light intensity changes and reduce the number of pairs during statistical analysis.

### Microscopic analysis of mycorrhiza

The intensity of mycorrhizal colonization in potato roots was determined by microscopic analysis using the aniline blue staining/vinegar method for the visualization of fungal structures. The arbuscular mycorrhiza rate was quantified according to the Trouvelot et al. (1986) method. For each plant, 30 images were taken at 100 × magnification using a Leica LMD6 microscope (Leica Microsystems, Wetzlar, Germany). Parameters, i.e., the intensity of mycorrhiza (%M) and arbuscule abundance (%A) in the root system, were calculated with Mycocalc software (https://www2.dijon.inrae.fr/mychintec/Mycocalc-prg/download.html).

### Oxidative stress indicators: H_2_O_2_, ascorbate (ASC), total glutathione (GSH) and lipid peroxidation

H_2_O_2_ levels in plant roots and leaves were examined with the colorimetric method described by Junglee et al. (2014) without freezing immediately after tissue acquisition. Briefly, 150 mg of tissue was homogenized in 1 ml of 0.1% TCA for 5 min. Homogenate (0.25 ml) was added to the reaction mixture containing 0.5 ml of 1 M KI and 0.25 ml of 10 mM potassium phosphate buffer (pH 7.0). For every sample, a background color control was prepared in such a way that water (0.5 ml) was used instead of KI. All samples were protected from light. After centrifugation, clear homogenate (0.2 ml) was transferred to a clean tube and incubated in darkness for 20 min at 20°C. Each series of reactions consisting of three replicates was blanked with its background color controls. The absorbance was measured at 350 nm using a NanoDrop 2000 spectrophotometer (Thermo Fisher Scientific, Waltham, MA, USA).

The total pool of ascorbate (tot ASC) and reduced ascorbate (red ASC) were examined according to Gillespie and Ainsworth (2007). A total of 100 mg of plant leaf was homogenized in ice-cold 1 ml of 6% TCA and centrifuged for 5 min at 13,000 *g* and at 4°C. The clear homogenate used for the total ASC assay (200 μl) was mixed with 100 μl of 75 mM potassium phosphate buffer (pH 7.0) and 100 μl of 10 mM DTT (Sigma-Aldrich, Saint Louis, MO, USA). After a 10-min incubation at RT, 100 μl of 0.5% NEM was added and further incubated for 1 min. The homogenate used for the reduced ASC assay was mixed with 100 μl of 75 mM potassium phosphate buffer (pH 7.0) and 200 μl of water. Then, both samples were processed in the same way. The following compounds were added to each assay tube: 500 μl of 10% TCA, 400 μl of 43% H_3_PO_4_, 400 μl of 4% α-α′-bipyridyl, and 200 μl of 3% FeCl_3_. After incubation for 1 h at 37°C, the absorbance was measured at 525 nm using a Hitachi U-2900 spectrophotometer (Hitachi Ltd., Tokyo, Japan). Oxidized ascorbate (DHA, dehydroascorbate) was calculated from the difference between total and reduced ASC.

The total glutathione was examined with a kinetic method using a Glutathione Assay Kit (Sigma-Aldrich, Saint Louis, MO, USA). The reaction product (TNB) was measured colorimetrically at 412 nm using a SpectraMax iD3 microplate reader (Molecular Devices, San Jose, CA, USA).

Lipid hydroperoxides were checked colorimetrically with a PeroxiDetect Kit (Sigma-Aldrich, Saint Louis, MO, USA). The absorbance was measured at 560 nm using a SpectraMax iD3 microplate reader (Molecular Devices, San Jose, CA, USA).

### TAS-ELISA

Potato virus Y levels in the leaves and roots of virus-inoculated potato plantlets were examined using a commercial kit, i.e., ELISA Reagent Set for potato virus Y (Agdia, Inc., Elkhart, IN, United States). The analysis included a commercial positive control for PVY provided with the reagent set. Positive control was used as a standard to prepare a standard curve by two-fold serial dilutions. The range of absorbance, in which the reaction was linear, was used to quantify the virus concentration in plant tissues. The obtained values were relative and calculated based on the standard curve equation but without absolute assessment of viral particles. Values allowed us to determine the degree of change in the PVY level. The ELISA protocol was as follows. A total of 50 mg of leaves from the fifth node and the same amount of roots were homogenized in the general extract buffer (GEB) at a 1:10 ratio. Then, the leaf homogenate was diluted 10 times, and the root homogenate was diluted two times to fall into the linear reaction range. The assay was performed according to the manufacturer's recommendations. For each series of analyses, one of the standards and a negative control were processed along with the samples.

### Statistical analyses

Raw datasets were checked with the chi-square test for observations lying beyond the 75th percentile (Outliers package in R). These values were detected as outliers and removed from further analysis. The data were checked to follow a normal distribution with the Shapiro-Wilk W-test. The homogeneity of group variances was checked with the Levene's test. Two-way ANOVA was applied to check the effect of AMF inoculation on growth and stress parameters in healthy and virus-infected plants. Two-way ANOVA (type II) was calculated in R using the “car” package. A *post hoc* Tukey test was applied to check significant differences between variant pairs. Plots were prepared with R using the “ggplot2” package. In box plots, the following elements are shown: the medium line inside the box indicates the mean value and the whiskers present the standard deviation. Letters assigned to each box mark ANOVA groups. Groups not sharing letters are significantly different (*p* ≤ 0.05). A *t*-test was applied to check differences within pairs of observations for photosynthetic parameters. Calculations were performed using R (R Core Team, 2022).

## Results

### Symptoms of PVY infection in potato

PVY^N:O^-T1 strain infecting potato plantlets (cv. Pirol) under *in vitro* conditions was asymptomatic, as virus-positive plants were indistinguishable from healthy plants. During the pot stage of the experiment, the symptoms of PVY infection appeared on leaves and included a mosaic pattern, leaf crinkling, and stunting (Figure 1). The virus caused neither veinal necrosis nor tuber disease. Arbuscular mycorrhiza had no visible effect on the severity of expressed symptoms.

### Mycorrhiza level in potato roots

**Figure 2:**
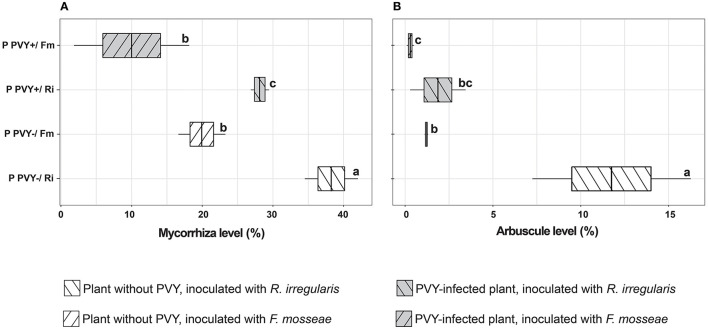
Level of mycorrhiza **(A)** and arbuscules **(B)** in healthy and PVY-infected potatoes after 12 weeks of incubation. The statistically significant differences are marked with letters. The same letters indicate no significant differences (*p* > 0.05).

The presence of AMF spores and hyphae on plant roots was checked at the end of the 12-week incubation. Before the microscopic evaluation of the arbuscular mycorrhiza level, we noticed many spores covering the root surface, which indicated the development of a symbiotic association between the host and fungi (Supplementary Figures 2, 3). Microscopic analysis revealed that the colonization level depended on the fungus used and the presence of PVY infection. After 3 months of plants' incubation, the average mycorrhiza level in PVY-negative plant roots was estimated to be slightly above 38% for *R. irregularis* and nearly 20% for *F. mosseae* (Figure 2A). The arbuscule amount for *R. irregularis* reached 11.8% vs. 1.2% for the other fungal species (Figure 2B). The observed differences were statistically significant, indicating that the two tested fungi colonized potato cv. Pirol roots to varying degrees. In PVY-positive plants, the root colonization level decreased by nearly 10% for each AMF species (for *R. irregularis* from 38.3 to 28.2% and for *F. mosseae* from 19.9 to 10%). At the same time, the arbuscule amount was four- to six-fold reduced, and the negative effect of the virus on plant-*R. irregularis* symbiotic association was stronger (11.8 vs. 1.9%).

### The effect of PVY and AMF on potato growth parameters

**Figure 3:**
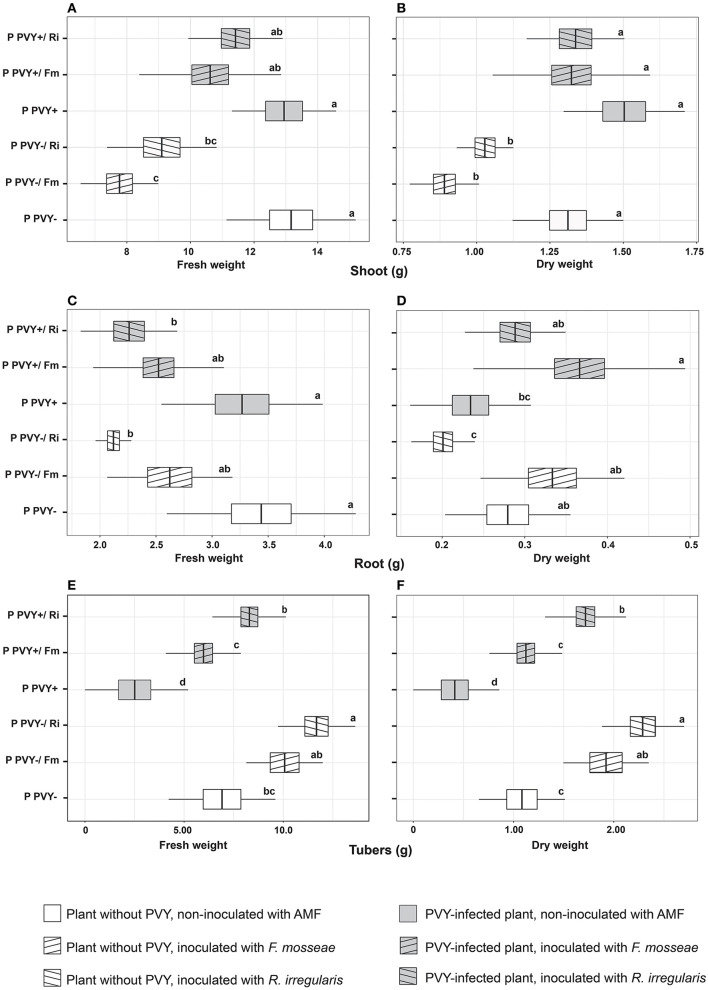
The combined effect of AMF inoculation and PVY infection on growth parameters of potato shoots **(A, B)**, roots **(C, D)**, and tubers **(E, F)**. The statistically significant differences are marked with letters. The same letters indicate no significant differences (*p* > 0.05).

**Figure 4:**
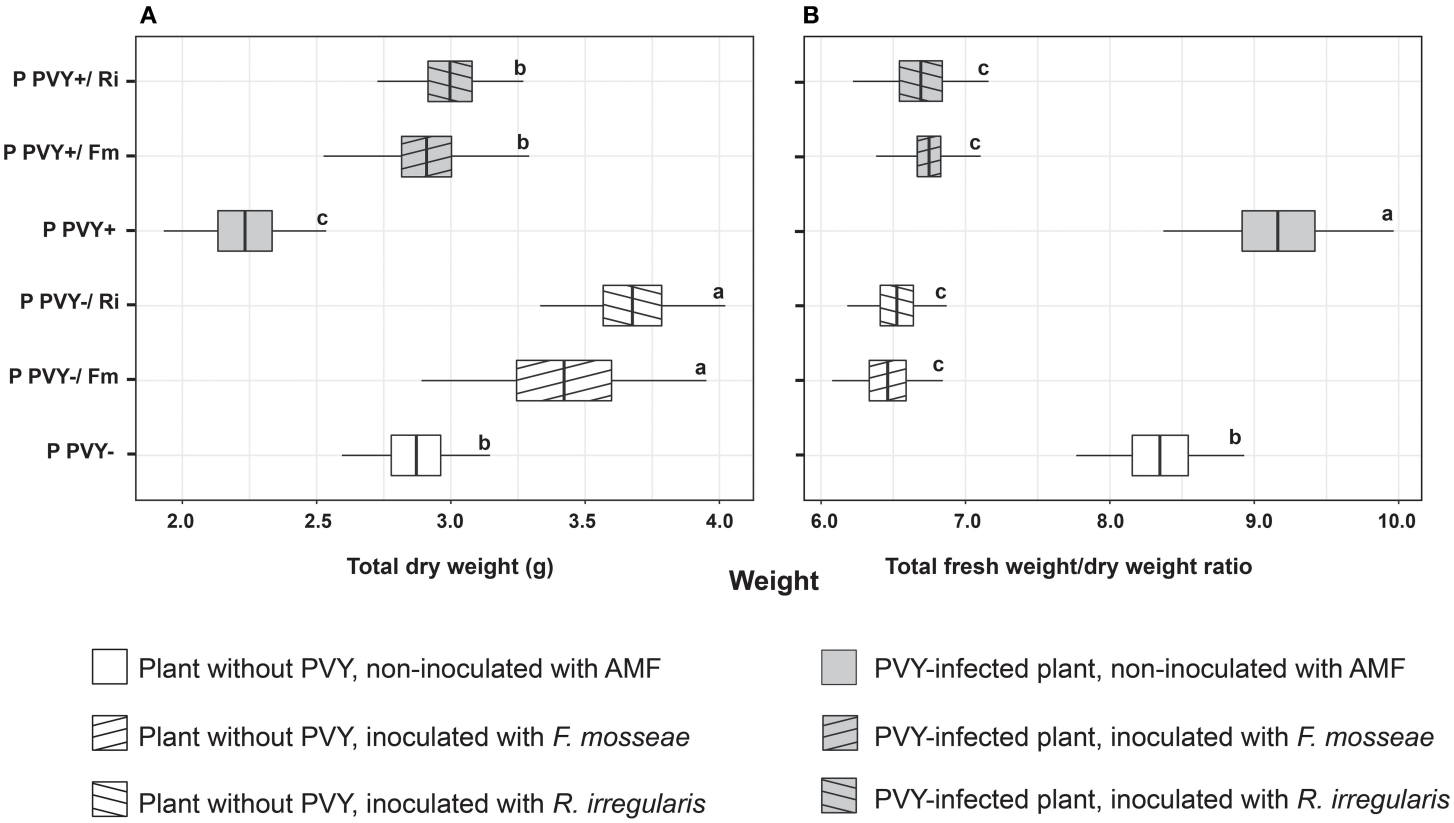
The combined effect of AMF inoculation and PVY infection on potato total dry weight **(A)** and ratio of total fresh to dry weight **(B)**. The statistically significant differences are marked with letters. The same letters indicate no significant differences (*p* > 0.05).

Potato virus Y itself had no influence on the shoot and root fresh and dry weight (Figures 3A–D). A significant effect of the virus was noticed for the fresh and dry weights of tubers, both of which were lowered by over 60% (Figures 3E, F). In the presence of PVY, tuber yield was dramatically decreased. Similarly, the virus significantly reduced the total dry weight of infected plants by 22.3% compared to healthy plants (Figure 4A). Interestingly, the presence of PVY in plants was associated with a higher total FW/DW ratio (Figure 4B), which showed that PVY-positive plants had a higher water content in tissues.

Surprisingly, AMF contributed to decreased shoot FW (*F. mosseae* by 41% and *R. irregularis* by 30.8%) and shoot DW (*F. mosseae* by 32% and *R. irregularis* by 21.4%) only in virus-free plants. When PVY infection and arbuscular mycorrhiza interacted with the host at the same time (variants PVY+/Fm and PVY+/Ri), plant shoot FW and DW did not significantly differ from those of the healthy control variant (PVY–; Figures 3A, B). The parameters of potato roots colonized with AMF depended on fungal species, as *F. mosseae* and *R. irregularis* acted differently. The first species caused a drop in root FW by ~23% for both variants PVY– and PVY+; however, the result was statistically insignificant. When noninoculated variants PVY– and PVY+ were combined and analyzed with a t-test against joined variants PVY–/Fm and PVY+/Fm, the drop in root FW was significant (*p* = 0.00053). At the same time, *F. mosseae* elevated root DW by 19% (for PVY–) to 56% (for PVY+). Analysis of combined noninoculated and inoculated variants also revealed significance (*p* = 0.0008). *R. irregularis* caused a significant decrease in root FW (by 31%−38%), irrespective of virus presence, and a drop in root DW (by 23%), but only in healthy plants.

The positive interaction between plants and arbuscular mycorrhiza was strongly reflected by the FW and DW of tubers (Figures 3E, F). Inoculation with AMF improved both parameters, alleviating the negative impact of the virus on tuber development (2.8-fold yield loss in PVY+ compared to PVY– plants). The effect of *R. irregularis* was more pronounced and significant each time, causing up to a three-fold increase in tuber FW and up to a four-fold increase in tuber DW, especially for PVY-infected plants.

Irrespective of AMF species colonizing potato roots, arbuscular mycorrhiza significantly improved the total DW of plants by 19%−34.5% (Figure 4A), which resulted in a decreased total FW/DW ratio (Figure 4B).

### The effect of PVY and AMF on oxidative stress in potatoes

**Figure 5:**
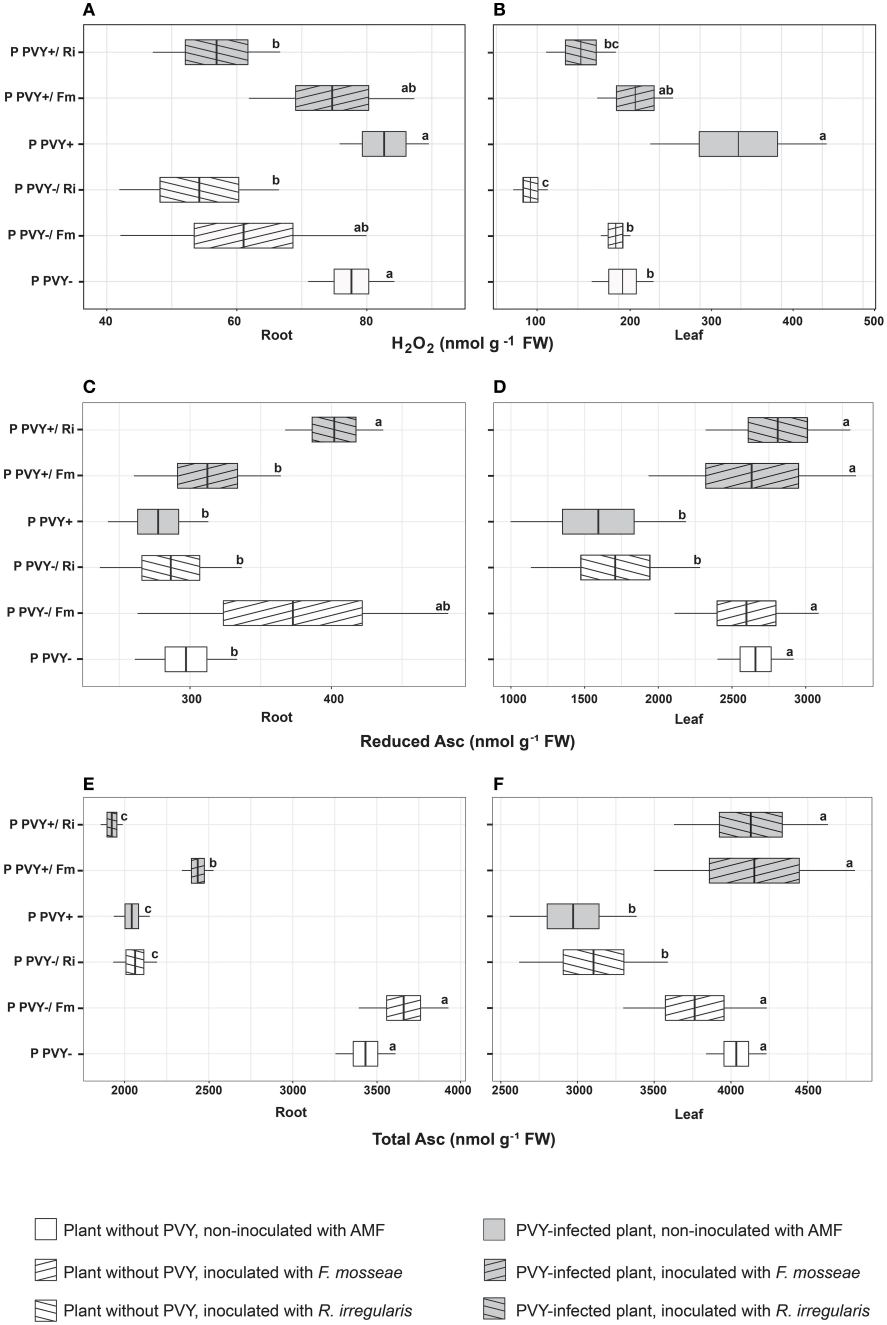
The combined effect of AMF inoculation and PVY infection on oxidative stress indicators—hydrogen peroxide **(A, B)**, reduced ascorbate **(C, D)** and total ascorbate level **(E, F)** in roots and leaves of in healthy and virus-infected potatoes. The statistically significant differences are marked with letters. The same letters indicate no significant differences (*p* > 0.05).

**Figure 6:**
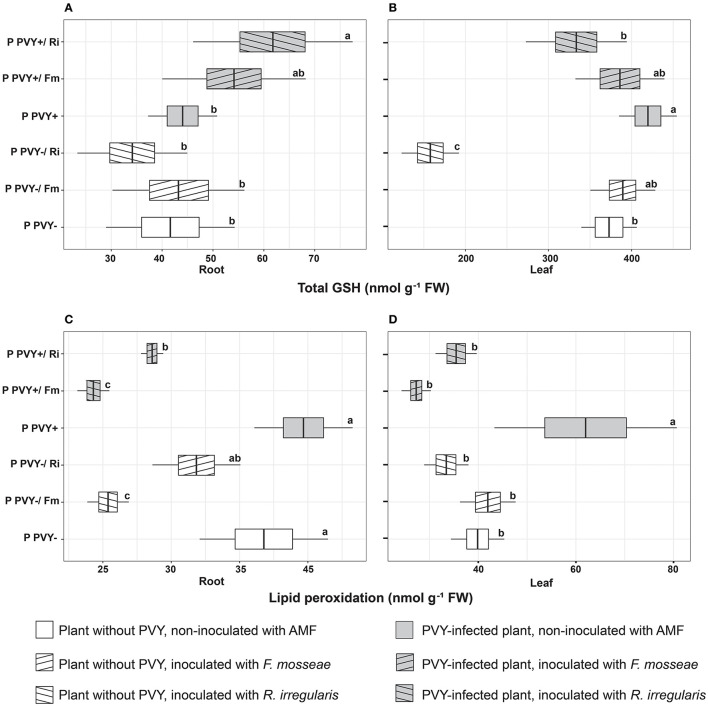
The combined effect of AMF inoculation and PVY infection on oxidative stress indicators in healthy and virus-infected potatoes. Measured parameters included total root and leaf glutathione **(A, B)** and lipid peroxidation level in root and leaf **(C, D)**. The statistically significant differences are marked with letters. The same letters indicate no significant differences (*p* > 0.05).

In the absence of arbuscular mycorrhiza, PVY seemed to exert a minimal effect on potato roots. The virus did not influence the H_2_O_2_ concentration (Figure 5A), total GSH content, or lipid peroxidation level (Figures 6A, C) in this plant organ, yet caused a dramatic decrease in the total ascorbate level (by 40.5%). However, it did not affect the reduced ascorbate pool, which was maintained at the same level as in healthy plant roots (Figures 5C, E). PVY induced different reactions in plant leaves. Virus presence caused significant elevations in the following parameters: leaf H_2_O_2_ concentration (by 69.4%), total leaf GSH content (by 12.6%), and leaf lipid peroxidation (by 55.4%) (Figures 5B, 6B, D). At the same time, PVY evoked a decrease in leaf total and reduced ascorbate levels (by 26.4 and 40%) (Figures 5D, F).

Potato colonization with *F. mosseae* did not affect the total concentrations of H_2_O_2_ and GSH, both in roots and leaves, irrespective of the absence or presence of viral infection. Some tendencies were observed; however, the final result was not significant. A minor positive effect of this AMF species was observed for the ascorbate level in PVY-positive plants. Arbuscular mycorrhiza increased the total ascorbate concentration in roots (by 19.2%) and leaves (by 39%) and at the same time elevated the pool of reduced ascorbate in leaves (by 65.5%). Importantly, *F. mosseae* significantly lowered lipid peroxidation levels in the roots (by nearly 39%) and leaves (by 56%) of virus-infected plants.

*Rhizophagus irregularis* seemed to exert stronger and more diverse effects on oxidative stress parameters in both healthy and PVY-infected potato cv. Pirol. In healthy plants, this species was shown to significantly decrease H_2_O_2_ (by up to 55%) and total ascorbate content (by up to 40%), for both analyzed organs. Additionally, inoculation with *R. irregularis* lowered pools of reduced ascorbate and total GSH in leaves only. This symbiont exerted no significant effect on the lipid peroxidation level, which was comparable to that of healthy control plants; however, a slight decrease in the value of this parameter could be detected (Figures 6C, D). In virus-positive plants, almost all observed effects of arbuscular mycorrhiza were significant (excluding total root ASC, which was maintained at the same level as in the control). After inoculation, lower levels of H_2_O_2_ and lipid peroxidation were noticed in the roots and leaves of PVY-bearing plants. The fold change in H_2_O_2_ was similar to that in virus-negative plants, while lipid peroxidation was reduced by 28%−42.7%. Similarly, the total GSH content in leaves decreased by nearly 20%. Contrary to these observations, other parameters, including the total ascorbate pool in leaves and total GSH content in roots, were increased by 39 and 36%, respectively.

### PVY and AMF effects on the photosynthetic capacity of potatoes

**Figure 7:**
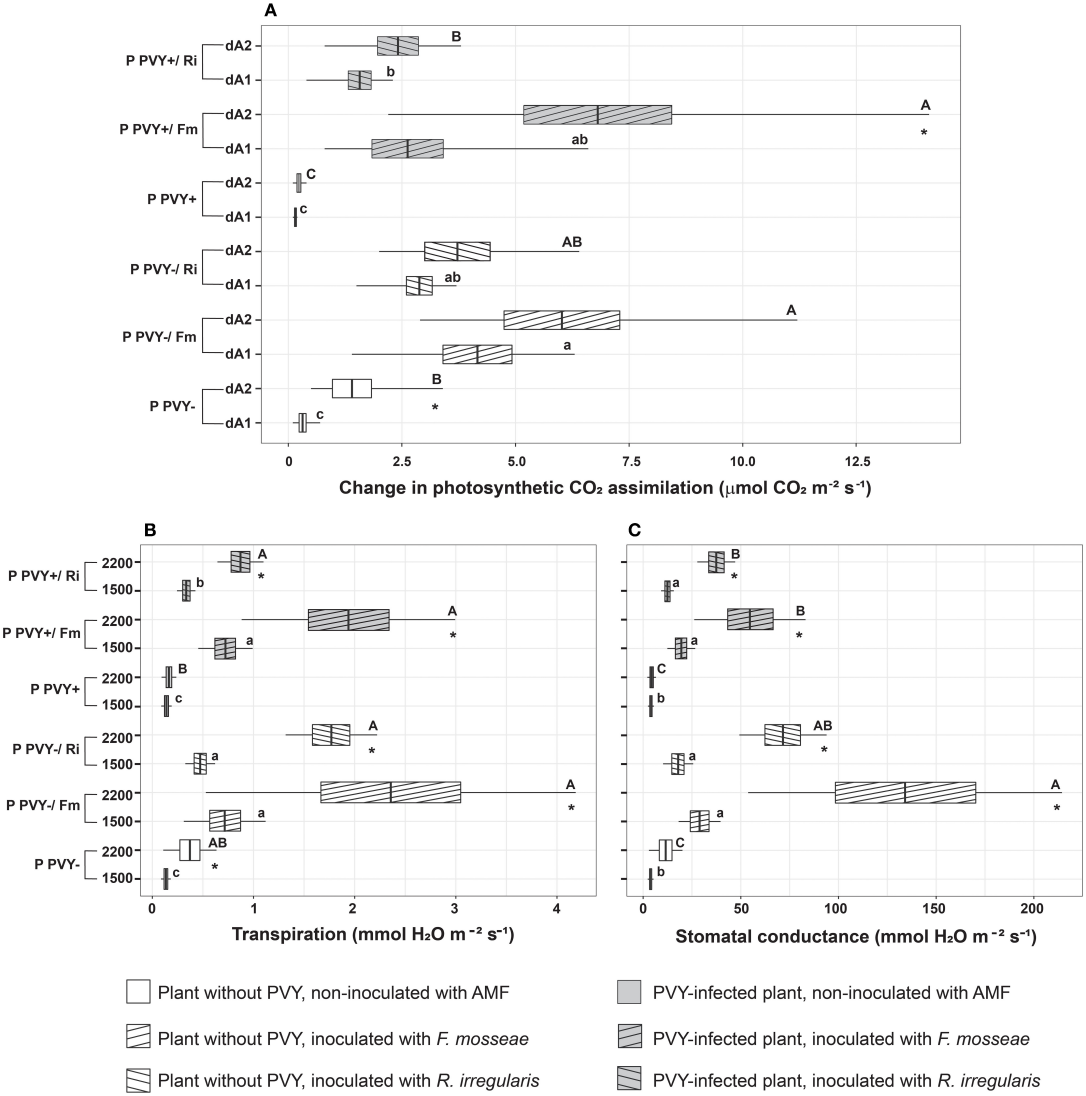
The combined effect of AMF inoculation and PVY infection on photosynthetic capacity in healthy and virus-infected potatoes. Measured parameters included CO_2_ assimilation rate **(A)**, transpiration **(B)**, and stomatal conductance **(C)**. Values dA1 (A_800_-A_500_) and dA2 (A_1,200_-A_500_) show changes in the assimilation rate between two different light intensities. The statistically significant differences are marked with letters. The same letters indicate no significant differences (*p* > 0.05). The asterisk means significant difference between each two parameters measured for the experimental variant (for **A**: dA1 and dA2; for **B, C**: 1500 and 2200).

Potato virus Y impacted plant parameters related to photosynthesis. A negative effect of the virus was observed for the CO_2_ assimilation rate, transpiration rate, and stomatal conductance at high light intensities exceeding 2,000 μmol m^2^ s^−1^ (Figures 7A–C). PVY-infected plants showed a very weak reaction (value dA2) to light intensity change (only a 1.5-fold increase) compared to healthy plants (a 4.5-fold increase).

Inoculation of roots with AMF, irrespective of fungal species, significantly improved the photosynthetic capacity of both healthy and virus-bearing plants. Positive effects were noticeable for both species and all photosynthetic parameters. *Funneliformis mosseae* seemed to exert a slightly stronger influence on plant photosynthetic capacity than *R. irregularis*; however, most of the differences were statistically insignificant and could be treated at best as a trend. Interestingly, all plant variants mycorrhized with *F. mosseae* showed the most pronounced increase in the dA2 parameter, reaching the same level of photosynthesis regardless of virus presence (a 4.3-fold increase for healthy plants and a 29-fold increase for infected plants). PVY+/Fm plants regained their photosynthetic capacity as they reacted comparably to PVY–/Fm. Similarly, the transpiration rate in virus-positive individuals was restored to the level measured in healthy individuals upon interaction with *F. mosseae* (reaching a value of nearly 2 mmol H_2_O m^−2^ s^−1^ at a light intensity of 2,200 μmol m^2^ s^−1^). At the same time, stomatal conductance followed the same trend; however, the effect of *F. mosseae* was the strongest in virus-free plants.

### The effect of AMF on PVY level in potatoes

**Figure 8:**
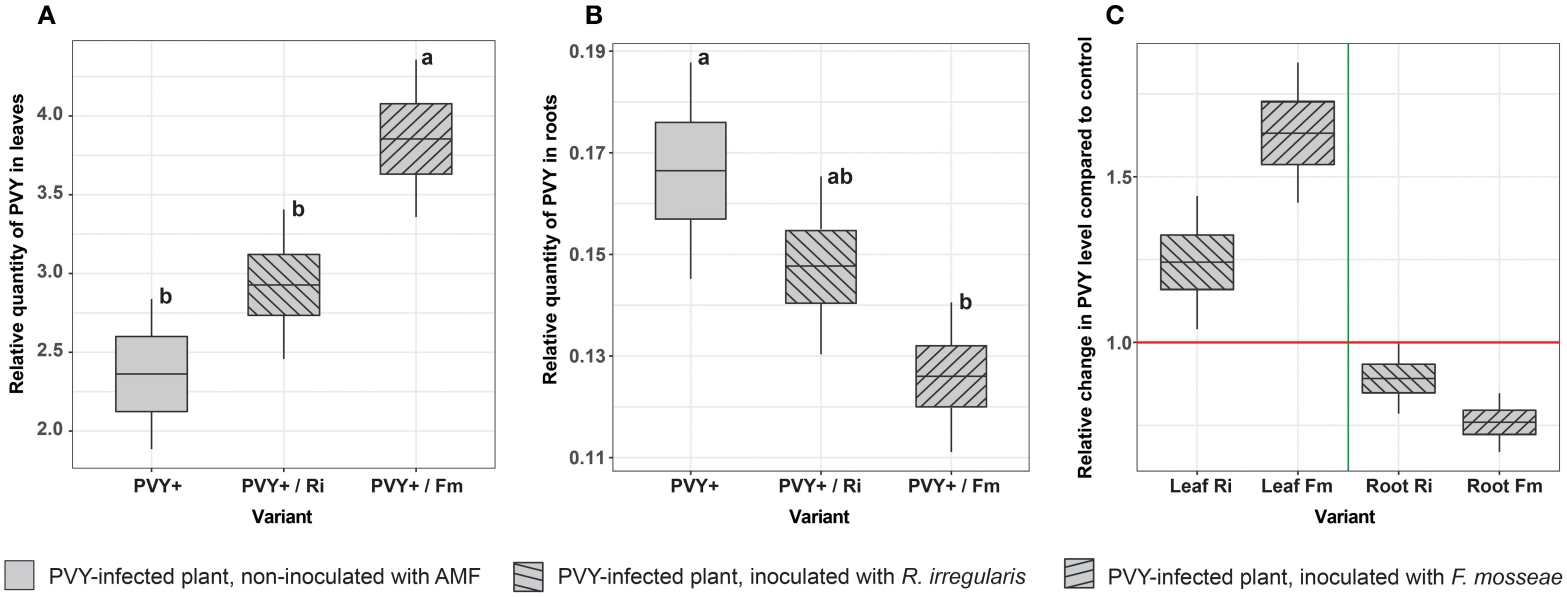
Effect of AMF on the relative quantity of PVY in leaves **(A)** and roots **(B)** in virus-infected potatoes. Each value is calculated as a ratio of the PVY level in mycorrhizal plants to that in nonmycorrhizal (control) plants. **(C)** Shows the fold change in the PVY level between mycorrhizal and nonmycorrhizal plant roots. The statistically significant differences are marked with letters. The same letters indicate no significant differences (*p* > 0.05).

The PVY content in noninoculated plants (variant PVY+) differed depending on the plant organ and was 13 times higher in leaves than in roots (2.36 vs. 0.166, respectively), which was expected due to virus characteristics. In general, both AMF species influenced the level of viral particles in the host in a similar way (Figures 8A, B), causing an increase in the PVY content in leaves and a simultaneous decrease in the PVY content in roots. Nevertheless, the significance of the observed differences depended on the fungus interacting with the plant. For *R. irregularis*, we noticed only a tendency with no statistical significance, while *F. mosseae* exerted a significant effect on PVY content. This species caused a more than 1.5-fold increase in virus levels in plant leaves (from 2.36 to 3.86) and a 1.32-fold decrease in roots (from 0.166 to 0.126) compared to nonmycorrhizal plants (Figure 8C).

## Discussion

Under environmental conditions, plants can host many different types of microorganisms and biotic factors, including viruses. Thus, plant shape and environmental fitness are modulated by multiple interactions between endophytes and phytopathogens inhabiting specific organs of the host. In this study, we demonstrated that AMF and PVY had an impact on both their host and each other. This tripartite interaction resulted in changed potato growth parameters, reduced mycorrhiza levels in roots, and altered accumulation of viral particles in roots and leaves.

Interestingly, we found that PVY infection can negatively impact potato-*R. irregularis* association, causing a dramatic drop in the number of developed arbuscules. At the same time, a similar but less pronounced effect was found for potato-*F. mosseae* symbiosis. This observation suggests that the two strains of AMF could have different levels of susceptibility to PVY. It is also possible that PVY induced some changes in host plant roots that greatly disturbed potato-*R. irregularis* compatibility. Moreover, the tested AMF species had different capabilities to induce virus translocation from roots to leaves. These phenomena may be associated with the activation of specific plant defense mechanisms, which in turn affect virus concentrations in host organs. The conclusion is based on the altered accumulation of the virus, depending on AMF species colonizing host roots. We found that *F. mosseae* induced greater changes, i.e., a stronger decrease in PVY content in roots and simultaneously higher accumulation of pathogen in leaves. We compared our observations with the outcomes of other studies. The number of articles on plant virus–AMF interactions is limited. Furthermore, these studies involved different plant-virus-AMF models. Some previous reports indicated that AMF lowered virus concentrations in plant organs (Maffei et al., 2014). In other reports, AM fungi were associated with elevated virus levels (Daft and Okusanya, 1973; Miozzi et al., 2011). Our finding is consistent with the results described by Sipahioglu et al. (2009), where *R. irregularis* stimulated an increase in the PVY reproduction and accumulation rate in leaves of potato cv. Marfona. Moreover, *F. mosseae* also induced long-term accumulation of phytovirus (tomatoes spotted wilt virus) in leaves of other solanaceous plants, i.e., tomatoes (Miozzi et al., 2011). However, none of these studies evaluated the effect of AMF on virus content in plant roots, which are the target organ for arbuscular mycorrhiza. We demonstrated that AM-induced changes in phytopathogen levels occurred in both leaves and roots. On the other hand, the lower the concentration of the virus in roots, the lower the impact of this pest on the symbiotic plant-fungus association. Interestingly, several mechanisms were proposed to link virus accumulation with arbuscular mycorrhiza (Hao et al., 2019). One of them involves viral multiplication due to increased P supply in plants expressing mycorrhiza-specific phosphate transporters PT4 (Daft and Okusanya, 1973; Lacroix et al., 2017). Better nutritional status of the mycorrhizal plant can enhance plant tolerance to virus pressure by compensating for the harmful effects of the pathogen (Hao et al., 2019). The other mechanism may be related to the downregulation of specific defense factors, e.g., pathogenesis-related (PR) proteins, heat-shock (HS) proteins, and glutathione S-transferase (GST) in virus-infected mycorrhizal tomatoes as was reported by Miozzi et al. (2011). Authors noticed that reduced activity of defense proteins was linked to increased infectivity of the virus in tomatoes and corresponded to virus accumulation in plant leaves. Finally, enhanced multiplication of the virus in mycorrhizal plants may be related to AMF-dependent improvement in their photosynthetic capacity. It was shown that infection sites can function as photosynthetic carbon sinks (Herbers et al., 2000; Zanini et al., 2021) to provide resources for virus replication. Thus, more efficient assimilation of CO_2_ due to mycorrhiza could result in more photosynthates, which in turn may positively affect virus development in potatoes.

Contrary to the results of the *in vitro* experiment (Deja-Sikora et al., 2020), where the colonization of potato cv. Pirol with *R. irregularis* did not influence shoot and root fresh weights, the effect obtained in the pot experiment was different. We noticed no positive effect of the two tested AMF species on either shoot or root growth (i.e., FW and DW). Interestingly, *R. irregularis* seemed to induce a stronger decrease in the values of the mentioned parameters than *F. mosseae*. On the other hand, healthy and PVY-infected plants inoculated with AMF produced significantly higher tuber yields, reflected by increased tuber FW and DW, and the effect was greater for *R. irregularis*. Furthermore, the total DW of PVY-free and PVY-positive mycorrhizal plants was significantly enhanced compared to that of the control and did not differ between the tested AMF species. The obtained results showed that AM improved the growth capacity of potatoes, independent of virus presence, but the effect could be seen mostly for belowground parts of plants, especially tubers. Our finding is in agreement with other communications, indicating that AMF improved potato yield quality and quantity (Duffy and Cassells, 2000; Douds et al., 2007; Hijri, 2016). Hijri (2016), who performed 3-year field trials, reported that *R. irregularis*-treated potato seeds gave nearly 10% higher yields than noninoculated controls. Similarly, Douds et al. (2007) revealed a 10 to 20% higher potato yield after the application of *R. irregularis* inoculum. Finally, Duffy and Cassells (2000) concluded that the final effect of plant–AMF interactions depends on the species used and can result in either a yield increase or decrease, as host–fungus functional compatibility is an important factor modulating an outcome (Ravnskov and Jakobsen, 1995; Lone et al., 2020; Santander et al., 2021; Fritz et al., 2022). We expect that the gain in tuber biomass observed in our experiment resulted from the better nutritional status of plants associated with AMF, as indicated by Liu et al. (2018) and Yang et al. (2020). The authors showed that arbuscular mycorrhiza was involved in enhanced phosphorus, nitrogen, and potassium acquisition by potatoes. P and N are key for plant biomass development, while K is important for plant osmoregulation (Clark and Zeto, 2000).

Surprisingly, the positive effect of arbuscular mycorrhiza on potato yield can be dramatically changed by phytoviruses. Sipahioglu et al. (2009) observed an 85% drop in tuber weight only when PVY and *R. irregularis* were present at the same time. Potato cv. Marfona almost completely inhibited tuber development as a result of virus–fungus interactions. In our study, the PVY-infected potato cv. Pirol reacted positively to both AMF species. As expected, the virus itself disturbed tuber production causing more than two-fold yield loss. The application of *F. mosseae* elevated tuber biomass to the level reached by healthy plants, while inoculation with *R. irregularis* showed an even stronger effect. In addition, we noticed no symptoms of viral disease exacerbation. We conclude that the positive effect of AM on PVY-infected potatoes is directly related to lowered levels of oxidative stress and improved photosynthetic capacity in mycorrhizal plants.

We measured different indicators of oxidative stress to assess the physiological condition of plants colonized with PVY and AMF. In our experiments, *F. mosseae* seemed to have a minimal effect on the oxidation status in healthy plants, as only a slightly lowered concentration of root H_2_O_2_ was noticed. Nevertheless, this change corresponded to a decreased lipid peroxidation in root cells. Again, *R. irregularis* regulated H_2_O_2_ levels and elements of the host antioxidative system much more strongly. In the absence of PVY, *R. irregularis* significantly reduced root and leaf levels of H_2_O_2_. This entailed further changes comprising decreases in total GSH (leaf), total ascorbate content (leaf and root), and lipid peroxidation (root). The measured parameters suggest that AMF, especially *R. irregularis*, alleviated oxidative stress in potatoes. Interestingly, the two AMF species interacted with the same host with different intensities. The degree of induced changes varied, depending on the species used, which was also noticed in other plant-AMF models (Cao et al., 2020; Malicka et al., 2021; Guo et al., 2022).

In our experiment, host plants responded to PVY with enhanced accumulation of ROS in leaves. This phenomenon was previously reported by many authors (Otulak and Garbaczewska, 2010; Deja-Sikora et al., 2020; Lukan et al., 2020). Additionally, the virus negatively impacted the total ascorbate pools in leaves and roots and simultaneously increased the GSH content, but the difference was significant only in leaves. All changes in the antioxidative system induced by PVY seemed to disrupt the oxidative balance in host plants as strongly increased levels of lipid peroxidation in leaves were apparent. Similarly, antioxidative imbalance and a higher lipid peroxidation rate, which are typical for susceptible host–virus interaction, were described by García-Marcos et al. (2009) in *Nicotiana benthamiana* infected with PVY and PVX. Several studies have reported that the accumulation of ROS upon systemic viral infection can contribute to disease symptom development including mosaic spots and plant deformations resulting in abnormal growth (Riedle-Bauer, 2000; Díaz-Vivancos et al., 2008; García-Marcos et al., 2009). Increased generation of ROS in leaves may be specifically linked to disturbed chloroplast metabolism, i.e., elevated electron leakage from photosynthetic electron transport chains, resulting from the inhibition of PSI and PSII (García-Marcos et al., 2009). This suggestion is supported by our observation of the adverse effect that PVY exerted on photosynthetic efficiency in potato cv. Pirol. Furthermore, Kogovšek et al. (2010) announced that PVY could upregulate the expression of antioxidant system enzymes, i.e., ascorbate peroxidase (APX), glutathione peroxidase (GPX), glutathione reductase (GR), and glutathione S-transferase (GST), and the effect depended on virus aggressiveness and host genotype. In this study, we noticed significantly increased leaf GSH content, which suggests a rather mild virus–host interaction.

In contrast to previously described results, both AMF species showed more diverse and pronounced effects on PVY-infected potatoes. As leaves are a target for PVY and roots are a place where mycorrhiza is developed, plant antioxidative responses were noted in both organs. In general, potatoes reacted to the virus and AMF with decreased levels of leaf and root H_2_O_2_. Arbuscular mycorrhiza additionally contributed to the elevation of the total ascorbate pool in leaves, while *R. irregularis* also lowered the GSH content in these organs. In roots, AM seemed to specifically increase GSH levels and either total (*F. mosseae*) or reduced (*R. irregularis*) ascorbate content. Changes in the oxidation status of PVY-positive plants associated with arbuscular mycorrhiza resulted in greatly diminished lipid peroxidation in root and leaf cells, which indicates AMF-induced alleviation of virus-caused stress. Nonenzymatic antioxidants, i.e., ascorbate and GSH, are linked to each other *via* the ascorbate-glutathione pathway used by plants for H_2_O_2_ reduction in water (Noctor et al., 2012). These results may suggest elevated activity of APX and GR in mycorrhizal plants infected with PVY; however, further confirmation is needed.

Finally, we found that PVY negatively impacted photosynthetic parameters including CO_2_ assimilation rate, transpiration, and stomatal conductance during increasing light intensity. The weaker photosynthetic activity of infected plants is possibly related to the photosystem inhibition mentioned earlier. Nevertheless, mycorrhiza improved the photosynthetic capacity of potatoes to such a level that PVY-infected plants reached similar values of the abovementioned parameters as healthy plants. This finding is, in general, in agreement with numerous studies where AMF positively influenced photosynthesis and protected the photosynthetic apparatus, especially under different types of stresses (Chandrasekaran et al., 2019; Gavito et al., 2019; Mathur et al., 2019; Popescu and Popescu, 2022). However, direct improvement of CO_2_ uptake efficiency, transpiration, and stomatal conductance in mycorrhizal PVY-infected plants was not demonstrated. Some indirect evidence, based on chlorophyll content analysis, was previously described (Sipahioglu et al., 2009; Deja-Sikora et al., 2020). It was only shown that AMF-colonized healthy potato cv. Pirol had a higher chlorophyll content than the control, but no such result was found in the presence of the virus (Deja-Sikora et al., 2020). Sipahioglu et al. (2009) reported no significant difference in this parameter for mycorrhizal PVY-infected potato cv. Marfona. This means that chlorophyll content may not adequately reflect the photosynthetic capacity of mycorrhizal plants. More reliable results are obtained by recording the CO_2_ assimilation rate, which reveals that AMF significantly contributes to the improvement of the photosynthesis process. It is not clear what exact mechanism underlies this finding; however, disinhibition in PSI and PSII (in spite of virus presence) is concluded.

## Conclusion

This study indicated that multipartite interactions can take place in plant hosts inhabited by phytopathogens and endophytes. We demonstrated that plant viruses exerted a negative impact on arbuscular mycorrhizal development in host roots. At the same time, AM was associated with an increased rate of virus multiplication in host leaves, possibly due to improved plant nutritional status, lowered activity of defense proteins, or increased plant photosynthetic capacity. Arbuscular mycorrhiza positively affected tuber yield in potatoes, alleviating the negative effect of PVY; thus, the application of AMF inoculum can reduce economic losses caused by the virus. Finally, AMF contributed to a decrease in virus-induced oxidative stress and protected cell lipids from peroxidation. Interestingly, the two tested AMF species interacted with the host variety at different intensities, suggesting that plant–fungus compatibility may be critical for obtaining benefits.

## Data availability statement

Datasets are available on request. The raw data supporting the conclusions of this article will be made available by the authors, without undue reservation.

## Author contributions

ED-S was responsible for the original manuscript preparation, text review and editing, the design and maintenance of the pot experiment, analyses of plant growth parameters, analyses of oxidative stress indicators, photosynthetic activity measurements, ELISA, and statistical analyses. KW assisted with the *in vitro* cultures of plants and AMF and participated in analyses of the plant growth parameters. KH conceptualized the study, supervised, reviewed the manuscript, and was responsible for the funding acquisition. All authors read and approved the final manuscript.
