# Supplementary material for: AMF species do matter: Rhizophagus irregularis and Funneliformis mosseae affect healthy and PVY-infected Solanum tuberosum L. in a different way
Source: Front Microbiol. 2023 Apr 17;14:1127278. doi: 10.3389/fmicb.2023.1127278 (PMC10150075; doi:10.3389/fmicb.2023.1127278)
Supplement: Supplementary file 1 [file Data_Sheet_1.pdf]

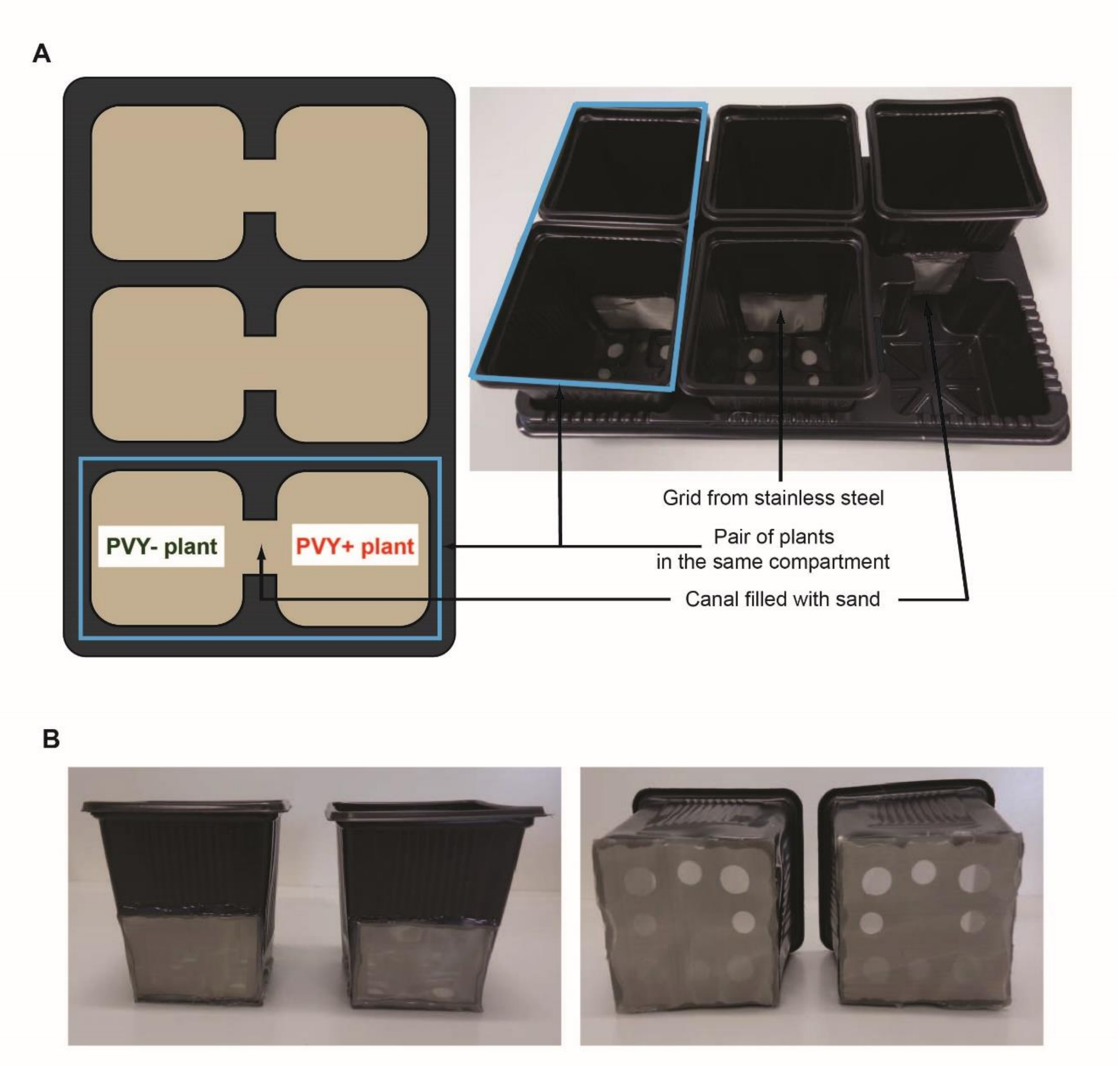

Supplementary Figure 1. Design of the tray for the pot experiment. (A) Each tray was divided into individual compartments containing pairs of plants. In each pair, one plant was healthy and the other was infected with PVY. (B) Pots from one pair had windows covered with a stainless steel mesh that allowed the development of mycorrhizal network.

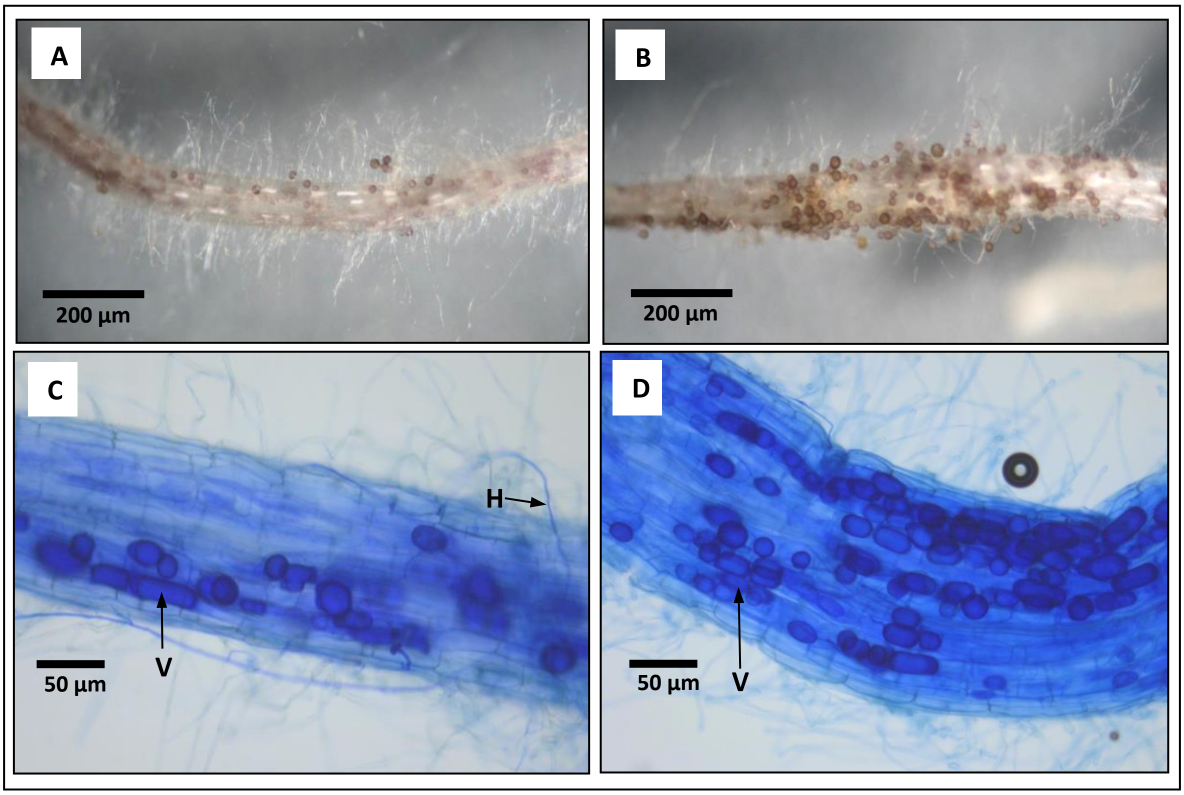

Supplementary Figure 2. Potato cv. Pirol roots colonized with AMF (A, C – *Funneliformis mosseae*; B, D – *Rhizophagus irregularis*). PVY-free roots covered with AMF hyphae and spores were observed at the end of experiment (A, B). Aniline blue staining of root fragments (C, D) showed AMF-specific structures inside the roots: V – vesicle, H – hyphae.

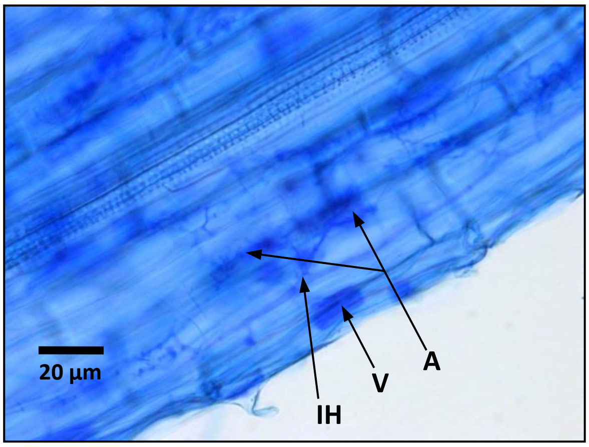

Supplementary Figure 3. Aniline blue staining of AMF-inoculated potato roots. Visible AMF structures: V – vesicle; A – arbuscule; IH – intraradical hyphae.
